# Use of nonhuman primates in obstructive lung disease research – is it required?

**DOI:** 10.5194/pb-4-131-2017

**Published:** 2017-06-30

**Authors:** Franziska Dahlmann, Katherina Sewald

**Affiliations:** 1 German Primate Center GmbH, Infection Pathology Unit, Kellnerweg 4, 37077 Göttingen, Germany; 2 Fraunhofer Institute for Toxicology and Experimental Medicine, Preclinical Pharmacology and Immunology, Biomedical Research in Endstage and Obstructive Lung Disease Hannover (BREATH), Member of the German Center for Lung Research (DZL), Nikolai-Fuchs-Straße 1, 30625 Hanover, Germany

## Abstract

In times of increasing costs for health insurances, obstructive lung diseases are a burden for both the patients and the economy. Pulmonary symptoms of asthma and chronic obstructive pulmonary disease (COPD) are similar; nevertheless, the diseases differ in pathophysiology and therapeutic approaches. Novel therapeutics are continuously developed, and nonhuman primates (NHPs) provide valuable models for investigating novel biologicals regarding efficacy and safety.

This review discusses the role of nonhuman primate models for drug development in asthma and COPD and investigates whether alternative methods are able to prevent animal experiments.

## 1 Introduction

Shortness of breath, wheezing, coughing, and chest tightness – these are the symptoms that affect 300 million people worldwide, with the reasons being obstructive lung diseases. Obstructive lung diseases do not only influence personal welfare, but they are also among the four most common causes of death worldwide, impairing both families and the economy (WHO, 2017). The collective term comprises the two diseases asthma and chronic obstructive pulmonary disease (COPD), which show increased prevalence at different stages in life. Whereas asthma is the most common chronic disease in children, the risk for COPD is increased later in life. To assess necessity of therapeutic intervention, both diseases are classified into different stages. The severity of asthma is classified according to the frequency of symptoms, lung function results, and requirements of therapeutics (National Asthma Education and Prevention Program, 2007). Similarly, COPD is categorized according to reduced lung function, the nature of the patient's symptoms, and the risk of exacerbation (GOLD, 2017).

Asthma symptoms occur due to airway obstruction either after an acute stimulus, e.g., an allergen, or as a result of chronic remodeling processes. In affected atopic patients, an acute asthmatic response occurs within minutes after allergen exposure, leading to reduced lung function due to airway obstruction, designated as early airway response (EAR). Mechanistically, allergens induce cross linking of mast cell bound immunoglobulin E (IgE), resulting in a release of mast cell mediators. These lead to airway obstruction by smooth muscle contraction, vasodilation, and increased vascular permeability. Several hours after this acute reaction, mast cell mediators induce an airway inflammation due to leukocyte influx in addition to mucus hypersecretion, which leads to another obstructive event, designated as late airway response (LAR; Galli and Tsai, 2012; Galli et al., 2008). Similarly, patients develop airway hyperresponsiveness (AHR) towards bronchial irritants, which is assessed in lung function analysis by methacholine exposure.

Repeated exposure to allergens leads to chronic inflammation, inducing structural remodeling processes in the airways. Inflammatory airway infiltrates are dominated by eosinophils accompanied by T lymphocytes, leading to epithelial cell injury. These cells are found in bronchoalveolar lavage (BAL) and biopsy material of asthmatic patients (reviewed in Howarth et al., 1994), associated with an increase in T helper (Th) 2 lymphocyte-derived immune mediators like the cytokines interleukin (IL)-4, IL-5, and IL-13 (Robinson et al., 1992; Huang et al., 1995; Kroegel et al., 1996). Remodeling processes include goblet cell metaplasia, subepithelial fibrosis, smooth muscle hypertrophy, and angiogenesis (Fahy, 2015), resulting in chronic airway obstruction.

Airway obstruction in COPD is a result of chronic exposure to irritants like smoke or air pollution. Continuous inhalation leads to inflammation of the airways, characterized by influx of primarily neutrophils; macrophages; and Th1, Th17, and cytotoxic T 1 (Tc1) lymphocytes. Whereas non-affected smokers develop only mild inflammation, smoking COPD patients develop an exaggerated inflammatory response, which remains after removing the irritant. The reason for this modified immune response is unknown, but genetic factors might contribute to disease outcome. Chronic bronchiolitis is accompanied by mucus hypersecretion and remodeling processes, including peribronchiolar and interstitial fibrosis resulting in airway obstruction. Chronic impairment of expiration, diagnosed in lung function analysis, leads to airway hyperinflation, thus inducing parenchymal destruction and emphysema formation (Barnes, 2016; GOLD, 2017).

Prevention of acute or chronically deteriorating symptoms requires ongoing therapeutic intervention both in asthma and COPD. Whereas mild and moderate asthma are well controlled by inhaled corticosteroids and long-acting beta-agonists (LABAs), severe asthma is largely therapy resistant. In these patients, alternative approaches involve application of therapeutic antibodies targeting IgE (omalizumab) and IL-5 (mepolizumab, reslizumab) (Brusselle and Bracke, 2014). Application of these antibodies significantly reduces exacerbations in severe asthmatics (Humbert et al., 2005; Ortega et al., 2014; Bel et al., 2014; Castro et al., 2015) but may not work if target antibody titers are above 3–4 % of total IgE (Johansson et al., 2009). Additionally, production costs are relatively high (Beck et al., 2010) and application is only approved in a clinical setting (EMA, 2009a) due to risk of anaphylactic reactions (reviewed in Khan, 2016).

Treatment of COPD patients requires ongoing treatment with long-acting muscarinic receptor antagonists (LAMAs) or LABA, which can be substituted with corticosteroids or other anti-inflammatory therapeutics, e.g., macrolides or phosphodiesterase-4 inhibitors. Whereas exacerbation can be reduced by inhaled glucocorticoids, they are not able to restore lung function capacity (Yang et al., 2012; Durham et al., 2016). Potential new targets include targeting the inflammasome, TNF-α (tumor necrosis factor alpha), IL-17 and IL-17 receptor, IL-18, and B cells (Brusselle and Bracke, 2014).

Overall, until today therapeutic approaches aim to prevent occurrence of symptoms and target structures which avoid amplification of existing inflammation. To prevent disease manifestation, causative mechanism of asthma and COPD need to be well understood in suitable animal models and human patients in translational approaches.

## 2 Analyzing obstructive lung diseases in nonhuman primates (NHPs) – does it make sense?

Ideal animal models for human diseases are those which naturally occur in an animal species, show identical pathology, and can be induced for direct availability. Obstructive lung diseases are mainly restricted to humans, whereas only a few animal species show naturally occurring diseases. Among these, horses and cats develop diseases similar to human asthma, and COPD-like symptoms appear in dogs (Williams and Roman, 2016). Nevertheless, recurrent airway obstruction in horses lacks eosinophilia and an acute airway response, while feline asthma shows comparable pathology to human asthma and is experimentally inducible (reviewed in Kirschvink and Reinhold, 2008). Dogs develop chronic bronchitis, while emphysema formation is only rarely observed (reviewed in Williams and Roman, 2016). Thus, among domestic animals the cat provides an attractive model for human asthma, but cross reactivity of human-specific biologicals might be absent for phylogenetic reasons.

**Figure 1:**
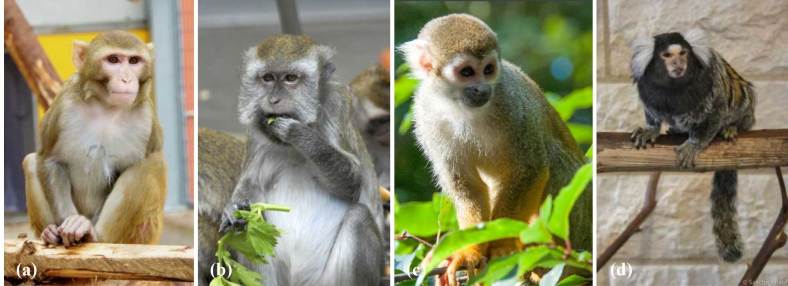
Nonhuman primates used in obstructive lung disease research. Rhesus macaques (*Macaca mulatta*) **(a)** and cynomolgus macaques (*Macaca fascicularis*) **(b)** belong to the family of old world monkeys **(a, b)**. Squirrel monkeys (*Saimiri sciureus*) **(c)** and marmoset monkeys (*Callithrix jacchus*) **(d)** are part of the new world monkey family. Source: **(a, b, d)**: German Primate Center GmbH (photographers: Margrit Hampe, JS Deutschland, and Sascha Knauf); **(c)** Serengeti Park Hodenhagen, Germany (photographer: Alexander Gail).

Due to close genetic homology, nonhuman primates provide targets for development of human-specific therapeutics in obstructive airway diseases. Classically, the old world monkey species *Macaca mulatta* (rhesus macaques) and *Macaca fascicularis* (cynomolgus macaques) are predominating species in obstructive airway disease research, but the new world species *Callithrix jacchus* (marmoset monkeys) and *Saimiri sciureus* (squirrel monkeys) have also been described (Hamel et al., 1986; Curths et al., 2015) (Fig. 1). Allergy-induced airway diseases have been observed in free-ranging macaques in Japan, which show rhinosinusitis after cedar pollen exposure (Hashimoto et al., 1994; Yokota et al., 1987; Sakaguchi et al., 1999). This indicates that macaques are generally susceptible to allergic reactions. Additionally, naturally *Ascaris suum*-sensitized macaques show positive skin prick tests and have been extensively used as inducible asthma models (Patterson and Harris, 1978; Gundel et al., 1990, 1992; Osborn et al., 1992; Turner et al., 1994; Mauser et al., 1995). To increase comparability to human allergic patients, further asthma models in nonhuman primates comprise experimental sensitization with allergens of house dust mites (HDMs, *Dermatophagoides pteronyssinus* and *Dermatophagoides farinae*; Yasue et al., 1998; Schelegle et al., 2001; Van Scott et al., 2004; Iwashita et al., 2008) and birch pollen (Bet V1 and Bet V2; Ferreira et al., 1996).

### 2.1 Old world monkey models of asthma

To allow testing and efficacy evaluation of human-specific biologicals, nonhuman primate models should comprise symptoms which are observed in human patients. Similar to humans, EAR can be observed in macaque asthma models after exposure to house dust mite (Van Scott et al., 2004; Iwashita et al., 2008; Schelegle et al., 2001), *Ascaris suum*, and birch pollen allergens (Ferreira et al., 1996; Patterson and Harris, 1978). LAR has been reported to occur after 4–8 h in HDM and *Ascaris suum*-induced asthma models (Gundel et al., 1992; Van Scott et al., 2004). Since airway hyperresponsiveness in humans is analyzed by exposure to the muscarinergic agonist methacholine, it is also a common technique in nonhuman primates (Madwed and Jackson, 1997; Van Scott et al., 2004; Iwashita et al., 2008). Independent of an allergen, histamine mediates bronchoconstriction in nonhuman primates, in contrast to rodents, highlighting the comparability to humans (Seehase et al., 2011; Schelegle et al., 2001; Patterson and Harris, 1978; Van Scott et al., 2004). Nevertheless it should be noted that airway reaction is usually assessed in anesthetized animals, in contrast to humans, and that anticholinergics typically used for premedication should be avoided in anesthetization to assess lung parameters.

Airway inflammation is assessed by BAL and endobronchial biopsies not as part of the routine asthma diagnostics but in human clinical studies, whereas BAL is a routine technique to evaluate inflammation in laboratory animals including nonhuman primates. In macaque asthma models, eosinophils are increased in animals, either sensitized with an HDM allergen or *Ascaris suum* (Gundel et al., 1990; Young et al., 1999; Schelegle et al., 2001, 2003; Miller et al., 2003; Van Scott et al., 2004; Iwashita et al., 2008). In addition, an increase in neutrophils has also been observed in some publications (Young et al., 1999; Schelegle et al., 2001; Van Scott et al., 2004), similar to an increase in CD4${}^{+}$ lymphocytes (Ayanoglu et al., 2011; Miller et al., 2003). Intervention with a glucocorticoid led to a shift in cellular composition, by reducing eosinophils (Van Scott et al., 2004; Ayanoglu et al., 2011; Plopper et al., 2012) and increasing CD8+ lymphocytes (Ayanoglu et al., 2011). Additionally, the combination of allergen and ozone led to a significant increase in eosinophils (Schelegle et al., 2003).

Bronchial biopsies are only rarely performed, since extensive pathological results are regularly obtained at the end of the studies. Biopsy material was investigated in two studies (Ayanoglu et al., 2011; Gundel et al., 1990), whereas only one study investigated pathological changes, which included eosinophils in lamina propria but did not reveal increased thickness of the basement membrane.

Pathological changes are similar to those observed in humans, although only reported in a limited number of animals (Schelegle et al., 2001; Van Scott et al., 2004; Joad et al., 2008). They include epithelial hypertrophy, goblet cell hyperplasia, thickening of the basement membrane, and eosinophilic infiltrations. Several experiments have been performed to analyze the impact of early sensitization on development of the airways and found a decrease in nerve fiber density in midlevel airways and a remodeled basement membrane zone, which could not be restored by reduction of allergen exposure (Larson et al., 2004; Kajekar et al., 2007; Evans et al., 2004). Additionally, smooth muscle cell mass was increased in infant macaques exposed to HDM early in life. Inflammatory infiltrations in HDM-challenged animals were significantly elevated, including increased CD1a${}^{+}$ dendritic cells and CD25${}^{+}$ T lymphocytes in epithelial compartments and increased CD1a${}^{+}$ dendritic cells, CD4${}^{+}$ lymphocytes, and IgE${}^{+}$ cells in interstitial compartments (Miller et al., 2005). Furthermore, mast cells were elevated in the extracellular matrix of the tracheal and intrapulmonary airways (Van Winkle et al., 2010).

Classical immune mediators have also been described in macaque asthma models. Like in humans, IL-4 and IL-5 showed increased levels in lung samples after stimulated peripheral blood cells of HDM-challenged monkeys (Miller et al., 2005; Iwashita et al., 2008; Van Scott et al., 2004). Further elevations were detected in BAL (IL-4, IL-5, IL-13), collected after inducing EAR, although these changes were only observed on gene level and not on protein levels (Ayanoglu et al., 2011). As a specific marker for sensitization, averaged HDM-specific IgE levels ranged between 6.3 and 9.6 U mL${}^{-1}$, in contrast to non-allergic animals with 2.5–3.1 U mL${}^{-1}$ (Ayanoglu et al., 2011; Van Scott et al., 2013). Similar to humans, four IgG subtypes have been described. In asthmatic patients, IgG4 is described as a biomarker, and the ratio of Ig subclasses is of increasing interest for specific immunotherapy (Kappen et al., 2017). In contrast to humans, cynomolgus macaque IgG2 and IgG4 show increased activity, which is counterbalanced by an elevated inhibitory activity of the FcRIIb receptor. IgG1 and IgG3 show almost identical binding activity and show cross reactivity with human FcγRI and FcγRIII receptors and vice versa (Warncke et al., 2012). Future studies will have to assess the behavior of IgG subclasses in macaque models to predict efficacy of, for example, specific immunotherapy.

In summary, old world monkey models of asthma are well established and share many features with human patients. Whereas some parameters still need to be assessed for better comparison to human patients, e.g., development of IgG subclasses, other parameters classically assessed in humans such as forced expiratory volume are not applicable in anesthetized animals. Nevertheless, future studies can help to understand disease mechanism, which cannot be studied in human patients in detail.

### 2.2 New world monkey models of asthma

In contrast to old world monkey models for asthma, pathological changes after sensitization and challenge of new world monkey models against a specific allergen have only rarely been described, although they are smaller in size, thus reducing the cost of performing a pharmacological intervention. Naturally sensitized squirrel monkeys have been used to evaluate changes in lung function after exposure to *Ascaris suum* antigen (McFarlane et al., 1984). Conscious but also anesthetized animals showed an increase in lung resistance and a decrease in dynamic compliance within minutes after inhalation, indicating the presence of EAR (McFarlane et al., 1984; Hamel et al., 1986). Similarly, LAR towards *Ascaris suum* was observed with a peak 4–9 h after initial exposure in four out of nine animals (Hamel et al., 1986). In a marmoset model of HDM-induced asthma, neither EAR nor airway hyperresponsiveness could be observed in sensitized, anesthetized, spontaneously breathing animals (Dahlmann et al., unpublished data), indicating differences in reactions towards naturally occurring allergens and induced sensitization.

Inflammatory infiltrations in marmoset monkeys can be either assessed by bronchoalveolar lavage or histopathology of the lung. BAL of HDM-challenged marmosets revealed increases in total cells, consisting of eosinophils, mast cells, and lymphocytes which were not further categorized (Curths et al., 2016). Macrophages and neutrophils were increased both in HDM- and control-challenged animals, indicating a nonspecific effect due to intratracheal application (Dahlmann et al., unpublished data). Pathological screening of lung tissue did not reveal an HDM-driven increase in inflammatory cells, but a nonspecific influx of inflammatory cells in control- and HDM-challenged marmosets (Dahlmann et al., unpublished data). However, differentiation of eosinophil and neutrophil infiltrates in marmoset lung tissue is challenging, suggesting the term “pseudoeosinophils” for this species (Bleyer et al., 2016) but complicating the characterization of this model. Further findings indicate a reduction in club cell secretory protein (CCSP) secreting cells in the airways, similar to human asthmatics who show reduced serum and BAL levels of CCSP (Van Vyve et al., 1995; Shijubo et al., 1999; Guerra et al., 2016) (Erffmeier et al., unpublished data). In contrast, goblet cell hyperplasia was absent, which might be attributed to differences in morphology of marmoset epithelial cells. Whereas the distribution pattern of CCSP-secreting club cells and goblet cells is similar between marmosets, macaques, and humans; the numbers of goblet cells are reduced in marmosets, and club cells represent the dominating non-ciliated cell type, in contrast to humans and macaques (Seidel et al., 2013). Other differences between humans and marmosets include only rarely observed submucosal glands (Seidel et al., 2013) and the occurrence of ciliated cells only in cartilage-free tracheal areas (Hoffmann et al., 2014).

Immune mediators of asthma have also been investigated in BAL and peripheral blood mononuclear cells (PBMCs) of HDM-challenged marmosets (Dahlmann et al., unpublished data). Among these, detection of many mediators failed (IL-2, IL-4, IL-5, IgE) or did not show group-associated differences (histamine, IL-17A, IFN-γ). However, HDM-specific IgG was detected only in sensitized and challenged animals, which was accompanied by HDM-induced release of IL-13 in PBMCs (Curths et al., 2015, 2016).

These data indicate that new world monkeys are generally suitable for induction of an asthmatic phenotype. However, one should consider restrictions due to limited characterization of the species and availability of reagents. Before evaluating a test substance, target cross reactivity and relevant endpoints should be carefully selected.

### 2.3 Monkey models of COPD

Only limited studies report COPD-like symptoms in old world and new world monkeys. As an inducer of neutrophilic airway inflammation, lipopolysaccharide (LPS) is frequently used in animal models to mimic COPD-like symptoms, since it is a contaminant of cigarette smoke or air pollution. In macaques, it induces an influx of neutrophils and lymphocytes and increases TNFα, IL-6, and IL-8 in BAL fluid, which are sensitive to glucocorticoid premedication (Mitchell et al., 2010). Cigarette-smoke-induced changes did not reveal changes in lung function in macaques. However, airway inflammation was confirmed by the increase in total leukocytes counts in BAL, consisting of neutrophils and macrophages. Increases in lymphocytes were only observed after 12 weeks of exposure. Cellular influx was accompanied by elevation of the cytokines CCL2 and IL-8 as well as the neutrophil migration factor matrix metalloproteinase (MMP) 9, but not MMP12, which is increased in rodent models of cigarette-smoke-induced COPD (Polverino et al., 2015). Pathological changes included an increase in submucosal glands and mucin-positive cells and remodeling of small airways and vessels characterized by deposition of extracellular matrix. Emphysema formation was absent, probably due to exposure of only 12 weeks, thus lacking a pathological finding frequently observed in human COPD patients (Polverino et al., 2015).

In new world monkeys, only LPS-induced neutrophilic inflammation has been reported in marmosets (Seehase et al., 2012). LPS-induced neutrophilic inflammation was associated with increased airway hyperresponsiveness, which could be abrogated by salbutamol (Curths et al., 2014). Additionally, bronchoalveolar lavage revealed increases in total cells, dominated by neutrophils, and elevation of the inflammatory mediators TNFα and MIP-1β (macrophage inflammatory protein-1β; Seehase et al., 2012). This phenotype was sensitive towards dexamethasone and roflumilast pretreatment. Pathological scoring was not performed, and remodeling processes as observed in COPD patients cannot be expected from only one LPS challenge.

In summary, nonhuman primates develop pathological changes corresponding to human COPD patients. So far, only macaques have been investigated in a setting mimicking continuous exposure to cigarette smoke, displaying remodeling processes and an elevation of markers comparable to humans. However, further characterization is required for analysis of human-specific therapeutics targeting relevant COPD markers.

## 3 Past and future approaches for novel respiratory therapeutics in nonhuman primates

Development of novel therapeutic approaches requires proof of efficacy and toxicological evaluation of substances in appropriate animal models. Before testing in an animal species, target structures have to be analyzed for genetic homology to address efficacy in the most suitable species. Additionally, binding affinity of a test substance towards this target structure needs to be assessed. Novel test substances in asthma and COPD are often directed against immunologic targets. Among these, monoclonal antibodies are highly target specific, requiring a close homology in animal species for efficacy assessment, which is often tested in humanized mice or nonhuman primates.

Monoclonal antibodies are promising therapeutics which have been developed in nonhuman primates. Among these, IgE is an attractive target for therapeutic intervention. Human IgE-Fc has been shown to cross react with cynomolgus monkey effector cells (Saul et al., 2014), and the binding of human IgE to cynomolgus lung tissue potentially drives IgE-mediated histamine release (Ishizaka et al., 1970; Wichmann et al., 2016). Later approved anti-IgE omalizumab showed binding to cynomolgus IgE (Fox et al., 1996; Meng et al., 1996), and efficacy assessment of a human-specific anti-IgE vaccine showed a reduction of circulating IgE, indicating the general suitability to test anti-IgE therapeutics in nonhuman primates (Weeratna et al., 2016). However, preclinical safety assessment of omalizumab in nonhuman primates revealed an age-dependent reduction of platelets, which was not detected in human patients, limiting the general transferability of data obtained in nonhuman primates (EMA, 2009a; Martin and Bugelski, 2012).

Other asthma-approved monoclonal antibodies which have been preclinically analyzed in nonhuman primates include the anti-IL-5 antibodies mepolizumab (Hart et al., 2001) and reslizumab (Egan et al., 1999). Cynomolgus monkey IL-5 shows cross reactivity against humanized anti-IL-5 antibodies (Hart et al., 2001; Mauser et al., 1995). Treatment of *Ascaris suum*-induced airway eosinophilia was reduced by both antibodies, with sustained effects for several months (Hart et al., 2001; Egan et al., 1999). In contrast, development of the anti-IL-4 antibody pascolizumab, which had been tested for cross reactivity and safety in cynomolgus monkeys (Hart et al., 2002), was discontinued in phase II studies due to lacking effectiveness of targeting only IL-4 (Akdis, 2012).

Further monoclonal antibodies for asthma therapy targeting IL-4 receptor subunit α (dupilumab), IL-5 receptor subunit α (benralizumab), and IL-13 (lebrikizumab, tralokinumab) are currently under development in phase III studies (Tan et al., 2016; NIH, 2017; EMA, 2017). Whereas data for most of these monoclonal antibodies regarding nonhuman primate studies are not available, tralokinumab significantly inhibited BAL eosinophils and reduced AHR in a cynomolgus *Ascaris* model of asthma and also in a humanized mouse model (May et al., 2012). Reduction of BAL eosinophils in cynomolgus macaques was also observed in anrukinzumab (Bree et al., 2007), whereas human patients with persistent asthma did not improve upon treatment. In patients with mild atopic asthma, lung function improved by using anrukinzumab, but sputum eosinophils were not reduced (Gauvreau et al., 2011), indicating the need to carefully select patients and endpoints. In contrast to asthma, there are no approved monoclonal antibodies available for COPD therapy, for example, against IL-17; however, it is not known whether these would prevent airway neutrophilia satisfactorily (Brusselle and Bracke, 2014).

All of these approaches were investigated in old world monkeys. Nevertheless, new world monkey species also have to be considered if they show better cross reactivity than other species. For example, canakinumab only binds IL-1β (Ilaris^®^, Novartis Europharm Limited, Horsham, UK) of humans and marmosets, in contrast to rodents and cynomolgus macaques, although high sequence similarity was shown. Therefore, marmosets were used for preclinical studies (Rondeau et al., 2015; EMA, 2009b). Other therapeutics applicable in obstructive lung diseases could also be investigated in new world monkeys, since, for example, roflumilast reduces neutrophilic responses in marmosets (Seehase et al., 2012).

In summary, it is important to test novel therapeutics in nonhuman primate species if targets show cross reactivity in vitro. However, transferability of results is not guaranteed, neither from rodents to NHPs (Wang et al., 2013) nor from NHPs to humans (Gauvreau et al., 2011; Martin and Bugelski, 2012). To gain insight into substance reactivity and relevant readout parameters in the target species, as much effort as possible should be made to reduce animal studies to a minimum, e.g., by applying alternative models.

## 4 Alternatives to nonhuman primate studies in obstructive lung diseases

The development of widely accepted alternative models for respiratory diseases, in particular in the context of inhalation of harmful substances, has lagged behind other routes of administration. This is due to the complexity of the respiratory system and diversity of local and systemic responses.

Alternative methods in respiratory research include in vitro cultures of different cell populations (monoculture, co-culture), depending on the localization within the respiratory tract, and ex vivo approaches (e.g., parenchymal strips, isolated airways, isolated perfused lung and precision-cut lung slices). In vitro cultures have been reviewed previously (Hittinger et al., 2015) and are essential tools for early steps in drug development. However, regarding NHP only limited numbers of cell lines are available, for example the rhesus macaque lung epithelial cell line 4MBr-5. The isolated perfused lung enables testing of substance deposition ex vivo and has been developed in rodents and rabbits, with rare reports in human and nonhuman primates (Byron et al., 1986; Niven and Byron, 1988; Piacentini et al., 2008; Bleyl et al., 2010; Linder et al., 1996; Briot et al., 2009; Hebb and Nimmo-Smith, 1948; de Burgh Daly et al., 1975; Nahar et al., 2013). Nevertheless, isolated perfused lungs can only be maintained for several hours, limiting extensive use.

Alternative 3-D tissue models, such as precision-cut lung slices (PCLS) are a link between in vitro and in vivo models. PCLS are fresh tissue sections of the lung. They contain all cell types that are present at the time point of preparation and reflect the functional heterogeneity within the respiratory tract. The PCLS technique has the advantage of reproducible preparation of thin tissue sections of precise thickness from one single animal or human tissue donor. Internal controls, technical replicates, references, and a variety of different concentrations of drugs and other substances can be included and tested in one donor. Moreover, PCLS offer the potential of sampling at different time points up to 2 weeks and were established in many different species, including NHPs (reviewed in Sewald and Braun, 2013; Seehase et al., 2011). They are widely used in toxicology and pharmacology. The field of application of PCLS has broadened from simply testing functional responses like airway constriction to disease modeling including calcium signaling, early allergic responses, and viral infection responses.

**Figure 2:**
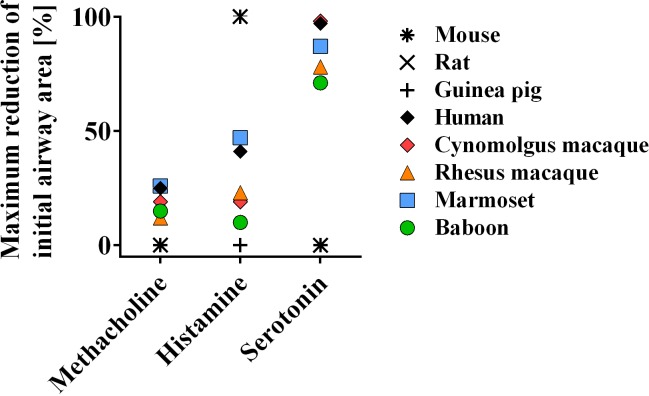
Nonhuman primates display bronchoconstriction similar to humans. Nonhuman primate and rodent PCLS were stimulated with methacholine, histamine, or serotonin, and the reduction of airway area was analyzed immediately. Adapted from Seehase et al. (2011).

**Figure 3:**
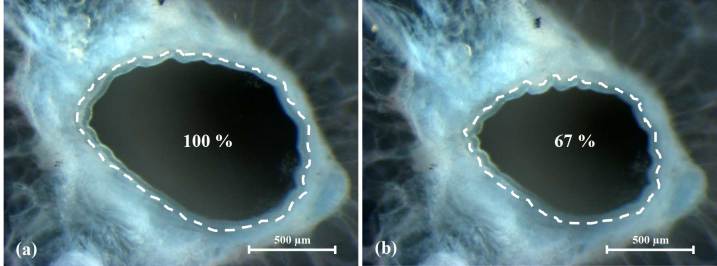
Cynomolgus PCLS of an ex vivo sensitized animal show bronchoconstriction after allergen exposure. Cynomolgus PCLS were passively incubated with 1 % human serum containing HDM-specific IgE. Reduction of the initial airway area (in %) was assessed by video microscopy before **(a)** and after exposure to HDM **(b)**. (photographer: Elaine Cabral Serrão).

NHP PCLS can be analyzed regarding bronchoconstriction and mediator release. Therefore, lung sections are prepared with cross-sectioned airways. Bronchoconstriction in PCLS can be induced by different mechanisms, e.g., by histamine independent cholinergic stimulation using methacholine, mimicking lung function analyses similar to in vivo settings (Fig. 2; Seehase et al., 2011). Moreover, PCLS derived from humans and NHPs can be passively sensitized with human plasma containing IgE against a specific allergen. Incubation of sensitized PCLS with the specific allergen results in bronchoconstriction similar to EAR, potentially by a mast cell associated mechanism, since increased histamine is detected after an allergen challenge (Wichmann et al., 2016) (Fig. 3). Antihistamines and disruptive IgE inhibitors prevent this bronchoconstriction also in NHP PCLS. Marmosets show the weakest reactivity towards a disruptive IgE inhibitor, potentially due to less cross reactivity of human IgE to marmoset FcεRIα (Saul et al., 2014; Wichmann et al., 2016). Comparing data of in vivo sensitized and ex vivo sensitized marmoset PCLS reveals a reduction of initial airway area to a similar extent (Dahlmann et al., Wichmann et al., unpublished data). Interestingly, the bronchial irritant capsaicin failed to induce bronchoconstriction ex vivo in marmoset PCLS (Schleputz et al., 2012) (Wichmann et al., unpublished data).

In contrast to humans and NHPs, serotonin (5-HT) but not histamine induces bronchoconstriction in rodents (Fig. 2). In another ex vivo approach, however, 5-HT induced a constriction in electric-field-stimulated tracheal rings of in vivo challenged rhesus macaques in a 5-HT${}_{2}$-, 5-HT${}_{3}$-, and 5-HT${}_{4}$-receptor-dependent, and 5-HT${}_{1}$-receptor-independent manner (Moore et al., 2012, 2014). These findings mimic the controversial discussion about the importance of serotonin in human asthma and highlight the importance of direct comparison of human and nonhuman primate material in an experimental setting.

Mitogens such as endotoxin induces an innate immune response in fresh lung tissue ex vivo. The released mediators are highly comparable to those released in vivo. Incubation of marmoset PCLS with LPS dose-dependently increased TNF-α and MIP-1β, which were inhibited by dexamethasone and roflumilast. Likewise, TNF-α and MIP-1β were elevated in BAL of marmosets challenged with LPS in vivo, and premedication with dexamethasone and roflumilast reduced TNF-α and MIP-1β release. Additionally, TNF-α secretion correlated between human and marmoset PCLS and roflumilast treatment resulted in a highly similar maximal inhibitory concentration (Seehase et al., 2012).

In conclusion, alternative approaches offer the advantage of direct comparison to human data and reproduce data obtained in vivo. Additionally, they help to identify suitable species for further safety testing of promising drug candidates

## 5 Conclusions

Overall, many protocols for inductions of obstructive lung diseases in NHPs have been investigated. Macaques especially show reproducible symptoms comparable to human patients, and different biologicals have been investigated in the *Ascaris* asthma model. Although macaques appear to be naturally sensitive to allergens, naturally occurring allergic asthma has not been reported, indicating potential limitations of macaque models. Future therapeutic approaches point towards an allergen-specific therapy, which might need adaptation of sensitization protocols in future efficacy studies. Additionally, endpoints have to be well considered and evaluated before study onset.

To improve a successful outcome of preclinical in vivo studies, an attempt should be made to exploit the full potential of in vitro and ex vivo assays, since they are valuable tools regarding certain endpoints. Besides a direct comparison to human data, they also help to reduce animal studies. However, they cannot mimic all endpoints, e.g., the complexity of immunologic reactions. Especially for novel therapeutics targeting human-specific immunological structures, NHPs are the only appropriate species due to sequence similarity and cross reactivity. Unfortunately, efficacy and safety in preclinical NHP studies does not guarantee efficacy and safety in clinical studies, highlighting the importance of well characterized in vitro, ex vivo, and in vivo studies.

In the future, precise characterization and categorization of obstructive lung diseases in human patients is also required, and identification of early biomarkers can help to prevent chronic disease progression. Only with novel approaches can improved animal welfare and a reduction of the worldwide disease burden of obstructive lung diseases be achieved – together we can make a difference.

## Data Availability

Data were not generated for this paper. All data which are designated as “unpublished data” are in progress for publication. For more information, please contact the contributing author.
